# Magnesium Sulfate as Adjuvant in Prehospital Femoral Nerve Block for a Patient with Diaphysial Femoral Fracture: A Randomized Controlled Trial

**DOI:** 10.1155/2018/2926404

**Published:** 2018-12-03

**Authors:** Chawki Jebali, Mohamed Kahloul, Nesrine Ibn Hassine, Mohamed Aymen Jaouadi, Fehmi Ferhi, Walid Naija, Naoufel Chebili

**Affiliations:** ^1^Emergency Medical Service, Sahloul Academic Hospital, Sousse, Tunisia; ^2^University of Medicine « Ibn Al Jazzar », Sousse, Tunisia; ^3^Department of Anesthesia and Intensive Care, Sahloul Academic Hospital, Sousse, Tunisia; ^4^Emergency Department, Ibn Al Jazzar Academic Hospital, Kairouan, Tunisia; ^5^Department of Anesthesia and Intensive Care, Farhat Hachad Academic Hospital, Sousse, Tunisia

## Abstract

**Introduction:**

Prehospital management of traumatic pain is commonly based on morphine while locoregional analgesia techniques, especially the femoral nerve block (FNB), can be safely and efficiently used. Adjuvants uses can reduce local anesthetic doses and decrease their related risk. The aim of the study was to assess the analgesic effect of magnesium sulfate when used as an adjuvant in prehospital FNB.

**Methods:**

This is a randomized double-blinded trial conducted in a prehospital medical department of an academic hospital. Patients with isolated diaphysial femoral fracture and eligible to participate were randomized into 2 groups. Group C had a FNB with 15 ml of lidocaine with epinephrine (300 mg) and 3 ml of normal saline solution. Group I had a FNB with 15 ml of lidocaine with epinephrine (300 mg) and 3 ml of MgS 15% (450 mg). The FNB was performed according to the WINNIE technique. Primary endpoints were morphine consumption and pain intensity during the first 6 hours. Secondary endpoints were the duration of the sensory block, time to the first analgesic request, and side effects occurrence.

**Results:**

Twenty-four patients were enrolled in each group. Both groups were comparable according to demographic characteristics, initial pain scores, and vital constants. In group I, morphine requirements were significantly lower (2 ± 2 mg versus 5 ± 3 mg, *p* < 10^−3^), analgesic onset was significantly faster, and the average time to the first analgesic request was longer (276 ± 139 min versus 160 ± 79 min, *p* < 10^−3^). The average duration of sensory block was longer in group I (226 ± 64 min versus 116 ± 70 min *p* < 10^−3^). No side effects were recorded.

**Conclusion:**

Magnesium sulfate should be considered as an efficient and safe adjuvant to lidocaine in prehospital FNB. This trial is registered with (NCT03597945).

## 1. Introduction

Traumatic pain is an independent risk factor of morbidity and mortality. It can lead to a decompensation of chronic diseases, a worsening of traumatic injuries, and an increase in blood loss through sympathetic activation. That is why, it is widely considered to be the fourth vital sign. Its management quality is one of the accreditation criteria of institutions in several countries.

The prehospital therapeutic arsenal is based traditionally on morphine at the expense of neurological and respiratory side effects, which can already worsen a precarious condition related to traumatic lesions leading to a poor patient prognosis.

Locoregional analgesia (LRA) techniques have been described to be safe and efficient in prehospital emergency medicine [1] especially the femoral nerve block (FNB) which can be achieved even in limited resources structures [2–4]. LRA-related risk can already be decreased by the aspiration test and by reducing the local anesthetic doses, what appears to be possible with adjuvants uses [5].

Because of its antagonistic effect on *N*-methyl-D-aspartate (NMDA) receptors and its role in the regulation of calcium influx into the cell, magnesium sulfate (MgS) seems to have a potential analgesic effect when used as an adjuvant in FNB [6–10].

The aim of our study was to investigate the analgesic effect of MgS when used as an adjuvant to FNB in prehospital medicine.

## 2. Methods

After approval by the Research Ethics Board, this randomized double-blinded clinical trial was carried out in the prehospital medical department of a Tunisian teaching hospital over a 3 years period (April 30, 2015, to April 29, 2018). All patients with isolated diaphysial femoral fracture were enrolled.

Inclusion criteria were age over 18 years and informed and writing consent. Exclusion criteria were body mass index over 30, fracture associated with vascular or sensory disorders, cardiovascular diseases, hepatic or renal impairments, neuromuscular diseases, opioids administration before the FNB, chronic pain, a long-term pain relief treatment, pretreatment with calcium or calcium antagonist, known allergy to one of the study drugs, infection at the injection site, open fracture and fracture undocumented by the imagery.

Based on the results of a previous study [5] and targeting a decrease of 1 cm in pain intensity assessed by the visual analogue scale (VAS), the sample size was assessed to be at least 22 patients in each study group, considering a threshold of 0.05 and a study power of 90%. We have increased our sample size in each group to 25 patients to allow possible dropouts.

Included patients randomly received, in a double-blind manner (using computer-generated allocation numbers sealed in brown envelopes), one of two local anesthetic solutions.

The control group (group C) had a FNB with 15 mL of 2% lidocaine with epinephrine (1 : 200,000) and 3 ml of normal saline solution.

The intervention group (group I) had a FNB with 15 mL of 2% lidocaine with epinephrine (1 : 200,000) and 3 ml of MgS 15% (450 mg).

All included patients had standard monitoring with noninvasive blood pressure, arterial pulse oxygen saturation, and an electrocardio scope. After secure peripheral venous access, 4 ml/kg/h of normal saline perfusion was administered.

Subsequently, a FNB was performed according to WINNIE technique [2]. In this approach, the anterior superior iliac spine and the pubic tubercle are marked and joined by a line. This connecting line corresponds to the inguinal ligament. The puncture site is 1 cm below the inguinal ligament and 1.5 cm lateral to the femoral artery. The needle is progressed until the tough resistance of the fascia lata is felt. The resistance is overcome by slightly increasing pressure. While cautiously advancing the needle further, there is often a second “loss of resistance” when the tip of the needle passes through the iliac fascia. Following the negative suction test, the local anesthetic solution is injected. A rigorous asepsia (surgical face mask and hair cap; proper hand hygiene; sterile gloves and drape kit; skin disinfection) was maintained during the whole procedure.

The FNB efficiency was evaluated 15 minutes later by the pinprick test. Pain was assessed by visual analogue scale (VAS) every 10 minutes for the first hour, then every 60 minutes until the 6^th^ hour after the block. Patients with a VAS >3 received morphine titration.

Primary endpoints were morphine consumption and pain intensity during the first 6 hours.

Secondary endpoints were the duration of the sensory block, time to the first analgesic request, and the occurrence of side effects (erythematic, sedation, decrease in average blood pressure, or heart rate of more than 15% of the initial basic value).

Data were collected on customized data collection sheets and analyzed using dedicated statistical software (IBM® Statistical Package for Social Science (SPSS), version 21.0, New York, USA). A *p* value < 0.05 was considered statistically significant. The continuous variables were expressed in average and standard deviation. The categorical variables were expressed in numbers and percentage. For the comparison of continuous variables, we used Pearson's Chi-2 test. For the comparison of categorical variables, we used Student's *t*-test. The two-way ANOVA test was used for comparison of VAS at different time periods of evaluation.

## 3. Results

**Figure 1:**
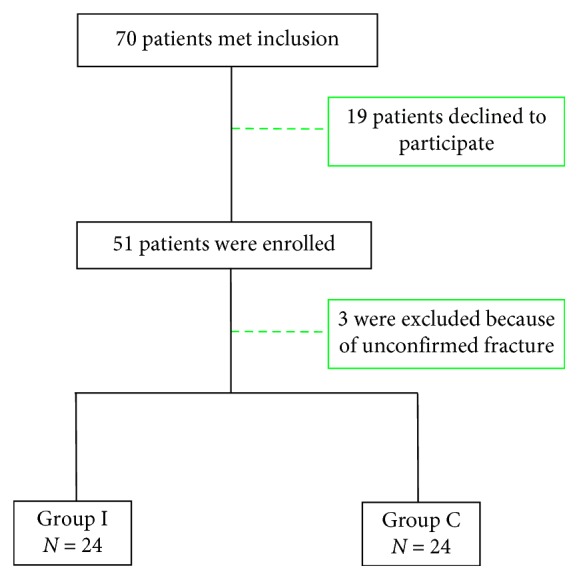
Flowchart of clinical trial.

During the study period, 70 patients met inclusion criteria. Nineteen patients declined to participate. Fifty-one patients were enrolled. Three patients were excluded because of unconfirmed fracture. Finally, 24 patients were enrolled in each group (Figure 1).

The average age was 66.5 ± 16.5 years. Both groups were comparable according to demographic characteristics, initial pain scores, and vital constants (mean blood pressure and heart rate) (Table 1).

**Figure 2:**
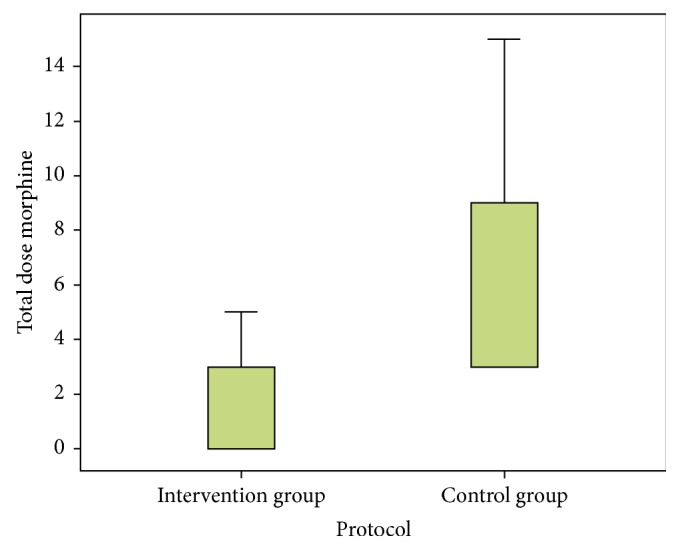
Morphine requirements in each group of the study.

**Figure 3:**
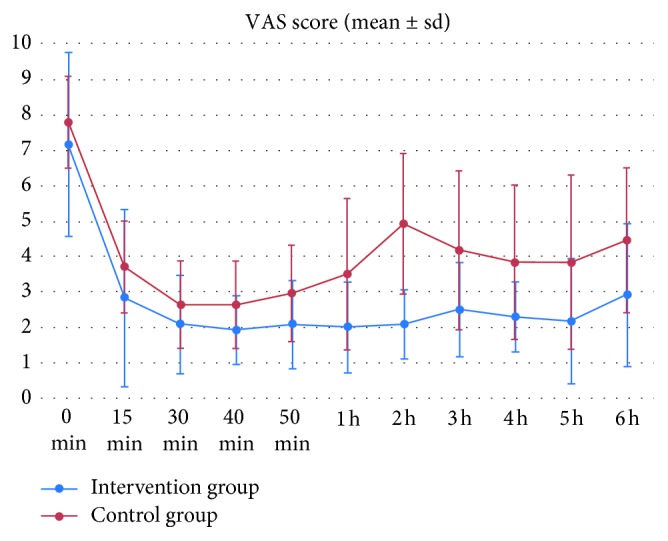
Evolution of pain intensity in both groups.

Morphine requirements were significantly lower in group I (median = 0 mg; IQI_25%–95%_ = [0–3] versus a median of 3 mg and a IQI_25%–95%_ of [3–9]; *p* < 10^−3^) (Figure 2). Analgesic onset was significantly faster in group I (Figure 3). The average time to the first analgesic request was longer in group I (276 ± 139 min versus 160 ± 79 min, *p* < 10^−3^). The average duration of sensory block was higher in group I (226 ± 64 min versus 116 ± 70 min, *p* < 10^−3^). No side effects were recorded during the study period.

## 4. Discussion

In this study, we found that the use of MgS as an additive to lidocaine in prehospital FNB for a patient with diaphysial femoral fracture improves analgesia quality. In fact, it reduces pain intensity with a rapid onset and a longer duration of time till the need for rescue analgesia. It also allows a reduction in morphine consumption without any side effects.

To our knowledge, this is the first report of MgS use in FNB in prehospital settings. This should encourage the use of LRA, in this context, as a safe and efficient analgesic technique especially for traumatic pain. The FNB is particularly appropriate for prehospital LRA as it is easy to perform even without sonography or neurostimulation. Thereby, it can be performed worldwide, even in limited resources structures. In addition to a pain-free transportation, it can also prevent pain accentuation related to the initial hospital management such as medical examination, radiological investigations, and some orthopedic procedures [3, 4]. Thus, an efficient, safe, and sustained analgesia should be provided, especially by adjuvants enhancing local anesthetics effects. In our study, patient with FNB without adjunction of MgS increased pain scores at hospital arrival. However, in the MgS group, analgesia profile was sustained without increasing morphine consumption.

Several trials have evaluated analgesic effects of MgS. Its perioperative intravenous infusion leads to a significant decrease in anesthetic requirement and postoperative analgesics consumption [8–11]. Postoperative analgesic effect was also reported with intrathecal administration. These actions seem to be due to its antagonistic effect on NMDA receptors [8, 12, 13] and its antagonistic effect on the calcium channel which inhibit the release of neurotransmitter in the synaptic cleft [6].

In a randomized clinical trial, 60 patients undergoing laser photocoagulation for varicose veins of the lower limb were enrolled. Then, they were assessed to receive FNB with bupivacaine (*n*=30) or FNB with bupivacaine and MgS (*n*=30). This study concluded that MgS adjunction provides a profound prolongation of block duration and a significant decrease in postoperative pain scores [14].

Several studies have evaluated MgS as an adjuvant in many LRA techniques such as spinal anesthesia, intra-articular administration, paravertebral block, and intravenous regional anesthesia [15–19].

According to Hwang et al. [20], MgS as an adjuvant during spinal anesthesia for patients undergoing total hip replacement improves postoperative analgesia but without significant differences with regard to postoperative need for rescue analgesia.

Bondok and Abd El-Hady [16] studied the effect of intra-articular MgS in knee arthroscopy. They found a significant decrease in postoperative pain scores and analgesic requirements.

Hassan and Mahran [18] conducted a study enrolling 90 female patients scheduled for radical mastectomy. Patients were randomized into two groups: group 1 received paravertebral block analgesia with bupivacaine alone and group 2 received paravertebral block analgesia with bupivacaine and MgS. Significant reduction in pain scores and postoperative opioid consumption were found in group 2.

Furthermore, Kashefi et al. [17] studied the effect of adding magnesium to lidocaine during intravenous regional anesthesia and found that magnesium sulfate enhances the analgesia quality without causing side effects. MgS interferes with peripheral nerves releasing neurotransmitters at the synaptic cleft and potentiates local anesthetics action [19, 21]. *N*-methyl-D-aspartate (NMDA) receptors have a major role in central nociceptive transmission, modulation, and sensitization of acute pain states [20]. Their activation leads to calcium influx into the cells, the action which can be blocked by magnesium [6]. The most likely acting mechanism seems to be a direct action of magnesium on the peripheral nerve by blocking the release of excitatory neurotransmitter at the synaptic junction or by potentiating the effect of local anesthetic [19, 22]. These analgesic effects were found with various doses of intravenous MgS ranging from 30 to 60 mg/kg with or without continuous infusion of 8 to 30 mg/kg/h [8–12,19,20]. Doses used in peripheral nerve blocks and intra-articular administration are about 500 mg [14, 16].

## 5. Conclusion

Magnesium sulfate should be considered as an efficient and safe adjuvant to local anesthetics in prehospital FNB. It allows a significant decrease in pain scores and opioid requirements without increase in adverse effects.

## Figures and Tables

**Table 1:** Comparison of patient characteristics between both groups.

	Group I (*n*=24)	Group C (*n*=24)	*p*
Age (years)	66.3 ± 23	64.2 ± 15	0.643
Gender			
Male	13 (54.2%)	11 (45.8%)	0.677
Female	11 (45.8%)	13 (54.2%)	
Personal history			
(i) Hypertension	13 (54.2%)	11 (45.8%)	0.677
(ii) Diabetes mellitus	15 (62.5%)	15 (62.5%)	0.245
(iii) Atrial fibrillation	0	2 (8.3%)	0.347
Circumstance of the accident			
(i) Falling	12 (50%)	14 (58.3%)	0.195
(ii) Traffic accident	12 (50%)	10 (41.7%)	
VAS-T0	7.17 ± 2.6	7.8 ± 1.3	0.215
Heart rate-T0 (beats/minute)	81.3 ± 10.3	80.5 ± 8.4	0.233
Mean arterial pressure-T0 (mm Hg)	88 ± 5.3	83 ± 6.5	0.07

## Data Availability

The data used to support the findings of this study are available from the corresponding author upon request.
